# Enumeration Agar, Acid Exposure and Sampling Time Are Relevant Factors Accounting for the High-Pressure Inactivation of Vegetative Pathogens in Fruit Puree

**DOI:** 10.3390/foods13162600

**Published:** 2024-08-20

**Authors:** Berta Torrents-Masoliver, Anna Jofré, Albert Ribas-Agustí, Sara Bover-Cid

**Affiliations:** IRTA (Institute of Agrifood Research and Technology), Food Safety and Functionality, Finca Camps i Armet s/n, 17121 Monells, Spain; berta.torrents@irta.cat (B.T.-M.); anna.jofre@irta.cat (A.J.); albert.ribas@irta.cat (A.R.-A.)

**Keywords:** *E. coli*, *L. monocytogenes*, *Salmonella* spp., high-pressure processing, infant food, microbial inactivation, sublethal injury

## Abstract

High pressure processing (HPP) is a non-thermal technology with emerging application within the fruit and vegetable sector. The impact of the enumeration agar on the recorded HPP inactivation of *L. monocytogenes*, *Salmonella* spp. and *E. coli* in banana–apple and apple purees was evaluated. Additionally, the HPP inactivation and sublethal injury was quantified in apple puree, considering the impact of acid exposure (24 h before HPP) and sampling time. Inoculated purees were pressurized at 300 MPa for 2 min. Enumeration was performed immediately and 24 h after HPP. HPP inactivation was 0.9-to-4.5-fold higher in apple than banana–apple puree. Compared with nutrient-rich media, selective agar enumeration overestimated the inactivation. HPP inactivation and sublethal injury of *L. monocytogenes*, *Salmonella* and *E. coli* was variable, mainly dependent on the exposure to acid and the sampling time. The 24 h-delayed enumeration slightly increased the inactivation. In apple puree, the CECT5947 strain of *E. coli* O157:H7 was the most piezo-resistant strain (1.5 log reduction), while *L. monocytogenes* Scott A was the most piezo-sensitive (6-log reduction when exposed to acid and sampled 24 h after HPP). All the studied factors should be taken into account when designing HPP treatments, performing product-specific validation studies and setting verification procedures.

## 1. Introduction

The safety of infant foods is of paramount importance, since they are intended for a very vulnerable population. Thus, manufacturers must guarantee the innocuity of their products through the application of suitable processing and preservation processes in the framework of pre-requisite programs and the Hazard Analysis and Critical Control Points (HACCP) [1]. Traditionally, infant fruit purees are submitted to thermal processing. However, consumers demand more minimally processed products with better preserved sensory properties and nutritional quality through environmentally friendly technologies. Therefore, non-thermal preservation technologies such as high-pressure processing (HPP) are gaining industrial acceptance among food producers, including the fruit and vegetable sector (e.g., fruit juices, vegetable dips), as HPP preserves health-promoting nutrients and bioactive compounds in fruit and vegetable matrices [2], making HPP a potential alternative to thermal processing of infant fruit puree.

HPP is usually applied as a batch process in which pre-packaged food is submitted to elevated pressure (usually 400–600 MPa for a maximum of 6–10 min for industrial application) to eliminate or reduce the load of pathogenic and spoilage microorganisms, ensuring food safety and extending shelf-life [3]. HPP has been shown to be a suitable technology to design control measures to inactivate the relevant bacterial pathogens in fruit juices [4,5], and public health authorities of several countries have recognized HPP as a listericidal treatment for ready-to-eat foods [6]. However, the efficacy of HPP depends on the microorganism (not only between species but also at the strain level, with a considerable impact of the physiological state), the intensity of the treatment and the food matrix characteristics [3,6,7]. In acid pH, vegetative bacteria are more vulnerable to high pressure, making HPP particularly interesting as a primary lethal treatment [8]. HPP may not always completely inactivate vegetative pathogens, but it may injure a proportion of the bacterial population, which may not always be capable of recovering to form a colony in the microbiological-counting media, particularly if it includes selective factors [3,5]. Despite several studies evaluating HPP inactivation of pathogenic *Escherichia coli*, *Salmonella* spp. and *Listeria monocytogenes* in different types of fruit juices having been published [9,10,11,12], no study has characterized the pathogen lethality of HPP in fruit purees intended for infants. Kultur et al. [13] assessed HPP as a potential alternative to thermal processing (pasteurization) in infant fruit purees, showing inactivation of total mesophilic aerobic bacteria, yeast and molds, while avoiding the formation of process-induced contaminants (e.g., HMF, furan, etc.).

Nowadays, only a few countries have national regulations or guidance documents for HPP processing, with performance criteria of at least a 5-log reduction of the target pathogen of concern [14,15,16], i.e., the most resistant microorganism/s of public health significance likely to occur in the food matrix. In the case of acid fruit products, these are *E. coli* O157:H7, *Salmonella* spp. and *L. monocytogenes*, the three of them with high tolerance to low pH, with minimum pH for growth reported to be 4.4, 3.8 and 4.4, respectively [17]. The Gram-negative enteric pathogens *E. coli* O157:H7 and *Salmonella* have caused several outbreaks associated with fruit and vegetable juices [18]. The Gram-positive *L. monocytogenes* is an increasing concern for ready-to-eat fresh fruits and vegetables, due to the prevalence and persistence along the food supply chain. It is the causative agent of invasive listeriosis associated with outbreaks involving different types of ready-to-eat fresh fruits and vegetables [19,20,21].

The main objective of this study was to evaluate the HPP inactivation and sublethal injury of *L. monocytogenes*, *Salmonella* spp. and *E. coli* in banana–apple and apple purees. Several enumeration strategies, including non-selective and selective agar media, were assessed. The impact of the exposure of the pathogens to the more acid matrix (apple) before HPP and the sampling time after the treatment were evaluated, as potentially relevant factors for setting and validating HPP conditions to achieve the given performance criteria, as well as for process verification through microbiological analysis of the treated product. The bacterial response was assessed by means of challenge testing of different strains of each species, first inoculated as a cocktail and subsequently as single strains, for a better understanding of the intraspecies variability.

## 2. Materials and Methods

### 2.1. Bacterial Strains

Four strains of *L. monocytogenes* (CTC1034 (serotype 4b), Scott A(4b), 12MOB047LM (GII) and 12MOB089LM (4b)), eight strains of *Salmonella* (CTC1003 (London), GN009 (Derby), GN0085 (Typhimurium) GN0082 (Enteritidis), GN001 (Enteritidis), GN002 (Typhimurium), CIP106188 (monophasic Typhimurium) and CCUG34136^T^ (Enteritidis)) and four strains of *E. coli* (LGM2092^T^ (O1:K1:H7), CTC1029 (O2), CTC1030 (O78) and CECT5947(O157:H7, non-toxigenic; stx2-)) were independently cultured in Brain Heart Infusion broth (BHI) (Beckton Dickinson, Sparks, MD, USA) for 8 h at 37 °C and subsequently sub-cultured in BHI for 18 h at 37 °C. Cultures were supplemented with 20% glycerol as a cryoprotectant and preserved frozen at −80 °C until use.

### 2.2. Fruit Puree Preparation and Characterization

Golden apples and bananas were purchased from a local retailer. Fruits were washed, manually peeled, and chopped. Fruits and 0.5% *w*/*w* ascorbic acid were blended in a homogenizer (Thermomix^®^TM31, Vorwerk, Wuppertal, Germany) until a homogeneous puree was obtained. To eliminate background microbiota that would interfere with the enumeration of pathogens, samples were hygienized by submitting to HPP at 600 MPa for 6 min in a Wave6000 industrial equipment (120 l-capacity, Hiperbaric, Burgos, Spain) and stored frozen at −20 °C until their use.

Titratable acidity (TA) was measured by titration to pH 8.2 endpoint and expressed as equivalents of malic acid [22]. Total polyphenol contents were assessed according to Singleton and Rossi [23]. Apple puree had pH 3.32 ± 0.03, 13.33 ± 0.06 °Brix, 0.68 ± 0.01% of malic acid and 2828 ± 194 mg/kg of total polyphenols, while banana–apple puree, prepared with apple and banana (1:1) had pH 4.02 ± 0.02, 15.47 ± 0.35 °Brix, 0.55 ± 0.02% of malic acid and 2091 ± 294 mg/kg of total polyphenols). Fruit purees were transferred into PA/PE plastic bags (Sistemvac, Estudi Graf S.A., Girona, Spain) and thermo-sealed without air.

### 2.3. Sample Inoculation

For experiments using a cocktail of strains, banana–apple and apple purees were 1% *v*/*w* inoculated with a cocktail of either *L. monocytogenes* (CTC1034 (serotype 4b), Scott A (4b), 12MOB047LM (GII)), *Salmonella* (GN085 (Typhimurium), CTC1003 (London) and CTC1754 (Rissen)) or *E. coli* (LMG 2092^T^ (O1:K1:H7) and CTC1029 (O2)) at an initial concentration of ca. 10^7^–10^8^ CFU/g. Cocktails of strains were prepared by mixing equal amounts of each strain after thawing the −80 °C culture.

For the experiments using single strains, apple puree was independently inoculated (1% *v*/*w*) with each strain listed in 2.1 at an initial concentration of ca. 10^7^–10^8^ cfu/g after thawing the −80 °C culture.

### 2.4. Treatments: Acid Expousre and High-Pressure Processing

Aliquots (ca. 5 g) of inoculated samples were packaged in PA/PE plastic bags and submitted to HPP immediately after inoculation (HPP samples). The treatment at 300 MPa for 2 min was carried out in a Wave 6000 equipment (120 l-capacity, Hiperbaric, Burgos, Spain). The pressurization fluid was water at an initial temperature of 19 °C. The come-up-time was 2.2 ± 0.2 min, while the decompression was almost immediate. Two independent experiments were carried out.

To evaluate the effect of acidity on pathogen inactivation, inoculated apple puree samples were subjected to acid exposure (AE samples). This process consisted of maintaining the inoculated samples at 4 °C for 24 h, during which the bacterial cells were in contact with the intrinsic acidity of the more acidic fruit puree (i.e., apple). Furthermore, to evaluate the combined effect of acid exposure followed by HPP (AEHPP samples), half of the AE samples were subjected to HPP as described above.

### 2.5. Sampling and Microbiological Analysis

Samples just after inoculation (without treatment) were analyzed as control samples to quantify the inoculum level. As microbiological analysis should be started as soon as possible upon receipt at the laboratory, preferably within 24 h as the maximum recommended time from sampling to microbiological analysis (e.g., transportation from the sampling location to the laboratory), microbiological analysis was performed in duplicate immediately after treatment and after a 24 h refrigerated storage after HPP.

Fruit puree samples were 10-fold diluted in buffered peptone water (Merck, Darmstadt, Germany), homogenized for 30 s with a vortex agitator, 10-fold serially diluted in saline solution and plated onto different enumeration media:

- (T): TSAYE (BD-Difco, Becton Drive Franklin Lakes, NJ, USA) as a non-selective rich recovery medium, widely used for the enumeration of bacteria, including sublethally injured cells [24];
- (TS): TSAYE+ 4% NaCl (*w*/*w*) as a selective agar not supporting the growth of HPP sublethally injured cells [25];
- (S): chromogenic agars as selective agar: CHROMagarTM *Salmonella* Plus (SPCM, CHROMagar, Paris, France) for *Salmonella*, CHROMagar *Listeria* (CHROMagar) for *L. monocytogenes* and REBECCA^®^EB agar (bioMérieux, Marcy-l’Étoile, France) for *E. coli*;
- (SO): the selective chromogenic agars with an overlay of TSAYE.

In all cases, agar plates were incubated at 37 °C for 24–48 h.

### 2.6. Data Analysis

Inactivation was computed as the logarithm of the ratio between cell counts of samples after HPP, AE and AEHPP treatments (*N*), and samples were enumerated just after the inoculation (*N_0_*) (1):

(1) $$Inactivation=\log\left(N/N_{0}\right),$$

Sublethal injury was estimated as the difference between log counts on TSAYE (T) and TSAYE + 4% NaCl (TS), which is equivalent to the logarithm of the ratio between the cell counts on T and TS (2):

(2) $$Sublethal\;injury\;(\log ratio)=log\left(\frac{{\left(CFU/g\right)}_{T}}{{\left(CFU/g\right)}_{TS}}\right)$$

The results of inactivation and sublethal injury were analyzed by ANOVA and Tukey’s multiple comparison test, and differences were considered significant at *p* < 0.05. The statistical correlation between inactivation and sublethal injury ratio was assessed through the linear regression analysis, estimating the slope, the determination coefficient (R^2^) and the *p*-value.

## 3. Results

### 3.1. Impact of Enumeration Agar and Sampling Time after HPP on the Bacterial Inactivation in Banana–Apple and Apple Puree

**Figure 1:**
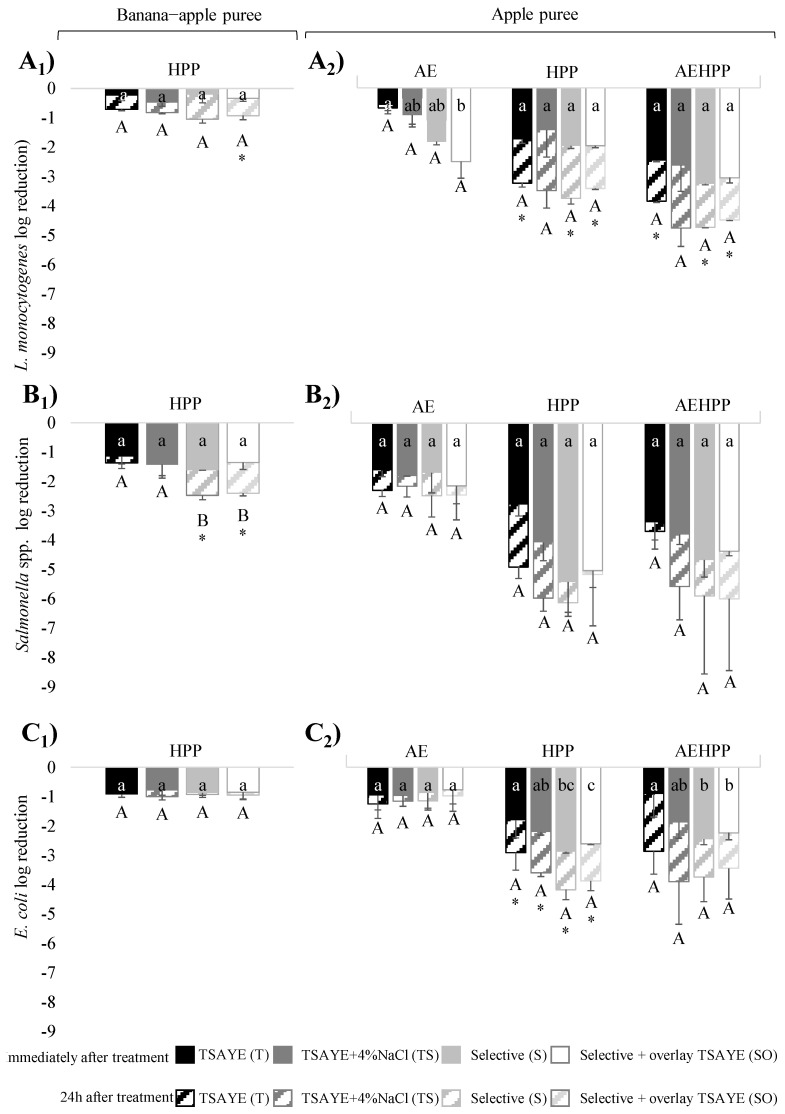
Mean logarithmic reductions of *L. monocytogenes* (**A**), *Salmonella* (**B**) and *E. coli* (**C**) strain cocktails in banana–apple (**A_1_**–**C_1_**) after high-pressure processing (HPP, 300 MPa for 2 min) and in apple puree (**A_2_**–**C_2_**) after acid exposure (AE), high-pressure processing (HPP, 300 MPa for 2 min) and both (AEHPP) treatments observed from the enumeration of survivors performed immediately after treatment (solid) and 24 h after treatment (striped). Error bars show standard deviation. Different letters show significant differences (*p* < 0.05) between treatments sampled immediately (lowercase letters) or 24 h (capital letters) after treatment. Asterisks show significant differences between the two sampling times.

Figure 1 shows the effect of HPP on *L. monocytogenes*, *Salmonella* and *E. coli* inoculated as a cocktail of strains in banana–apple and apple purees, according to the enumeration in non-selective (T) and different selective plate count agars (TS, S, SO). In banana–apple puree (Figure 1A_1_–C_1_), the effect resulted in an inactivation of 0.3 ± 0.2 log of *L. monocytogenes*, 1.4 ± 0.3 log of *Salmonella* and 0.9 ± 0.1 log of *E. coli* concentration when sampling was performed immediately after pressurization, the enumeration agar not significantly affecting the inactivation level for any of the evaluated species. A 24 h delay in the sampling time slightly increased the HPP inactivation shown by *L. monocytogenes* in SO agar (0.6 log more compared with sampling immediately after HPP) and *Salmonella* in S and SO agar (0.9 and 1.1 log more, respectively). At this sampling time, only *Salmonella* showed differences between agars, the inactivation obtained from S and SO agars being slightly higher (up to 1 log) than T and TS (*p* < 0.05).

In apple puree (Figure 1A_2_–C_2_), the three species showed higher HPP inactivation than in banana–apple puree (ranging from 0.9 to 4.5-fold) and, except for a few cases, in general no significant differences were detected between the four different enumeration agars. More specifically, *L. monocytogenes* inactivation in apple pure measured immediately after HPP was 1.8 ± 0.3 log on average for the different agars, and significantly increased to 3.5 ± 0.2 log when determined 24 h after HPP (*p* < 0.05). *Salmonella* was the most sensitive pathogen, showing similar log reductions when sampled immediately (4.3 ± 1.2 log) and 24 h after HPP (5.5 ± 0.7 log). In the case of *E. coli*, significant differences among the enumeration agars were observed when sampled immediately, with the inactivation ranging from 1.8 ± 0.3 log (with the non-selective T agar) to an average of 2.8 ± 0.2 log (with the selective chromogenic S and SO agars). The enumeration performed 24 h after HPP resulted in a significantly (*p* < 0.05) higher inactivation compared with that recorded immediately after HPP, showing an average of 3.7 ± 0.2 log inactivation reduction, without statistical differences among the enumeration agars.

Exposure of cells to the acidity (AE treatment) of the apple puree had a slight lethal effect on the three pathogens. Remarkable differences (*p* < 0.05) in the inactivation of *L. monocytogenes* were observed depending on the enumeration agar (from 0.5 log with TSAYE to 2.5 log with SO), without differences between immediate and 24 h-delayed samplings. In contrast, *Salmonella* and *E. coli* did not show significant (*p* > 0.05) differences in the inactivation by AE on different agars nor any due to delayed sampling. *Salmonella*, the most sensitive to AE, decreased by 1.7 ± 0.6 log on average, while *E. coli* was the most resistant to AE, with an average reduction of 1.0 ± 0.4 log.

The effect of acid exposure followed by HPP (AEHPP) depended on the bacterial species, enumeration agar or sampling time. For *L. monocytogenes*, similar inactivation was quantified with the different enumeration agars, but higher inactivation was observed 24 h after the AEHPP treatment (4.4 ± 0.5 log) compared with that observed immediately after the AEHPP (2.8 ± 0.5 log). The *Salmonella* cocktail did not show significant differences in the AEHPP inactivation measured with different agars, showing a mean inactivation of 4.0 ± 0.6 log and 5.2 ± 1.0 log after immediate and delayed sampling, respectively. However, the differences were not statistically significant, due to the large variability in the bacterial survival observed on the delayed sampling after AEHPP (as shown by the standard deviation). For *E. coli*, significant differences between T (0.9 ± 0.2 log reduction) and selective agars (S and SO agars, 2.4 ± 0.2 log reduction on average) were observed immediately after AEHPP.

### 3.2. Strain-Variability Inactivation by HPP and/or Acid Exposure in Apple Puree

According to the results described in Section 3.1, T and TS enumeration agar could recover a higher number of stressed cells after the assessed treatments, compared with the selective chromogenic agars. Therefore, the TSAYE-based agars were used to assess the variability of the inactivation caused by HPP, AE and AEHPP between different strains of *L. monocytogenes*, *Salmonella* spp. and *E. coli* in apple puree.

**Figure 2:**
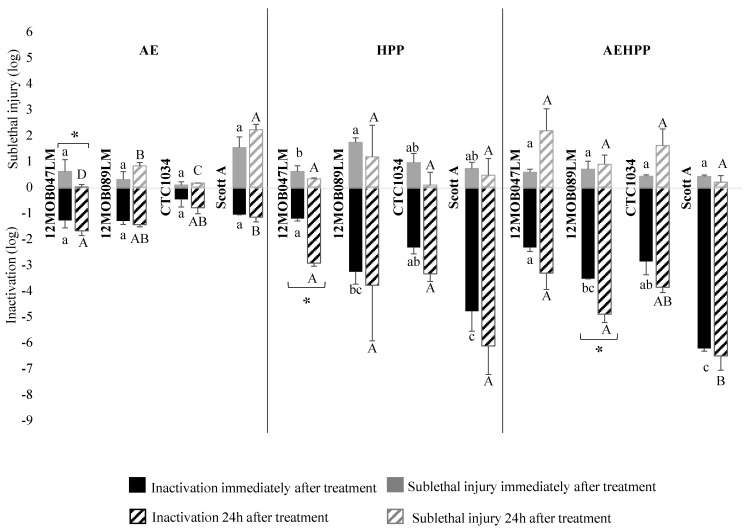
Mean logarithmic reductions (black) and sublethal injury (grey) for each strain of *L. monocytogenes* after acid exposure (AE), high-pressure processing (HPP) and both (AEHPP) treatments of apple puree sampled immediately (solid) and 24 h (striped) after treatment. The standard deviation is shown with error bars. The different letters show statistically significant (*p* < 0.05) differences between strains within each treatment (lower case for inactivation immediately after treatment, and upper case for inactivation 24 h after the treatment). Asterisks show statistically significant (*p* < 0.05) differences between sampling immediately and 24 h after treatment.

The inactivation of *L. monocytogenes* strains was variable (Figure 2). Inactivation due to AE was low, and although the CTC1034 strain was the most resistant to the apple pure acidity, the differences among strains were not statistically different. HPP inactivation ranged from 1.2 log for the most piezo-resistant strain (12MOB047LM) to 4.8 log for the most piezo-sensitive (Scott A). The combination of AE and HPP (AEHPP treatment) increased inactivation, in particular for the most sensitive strain, Scott A, to 6.2 ± 0.1 log; *p* < 0.05. A 24 h delay in the enumeration after treatments slightly increased the inactivation, though statistical significance was only confirmed for 12MOB047LM in HPP (1.0 log) and 12MOB089LM (1.4 log) in AEHPP.

The combined application of AE and HPP resulted in an inactivation equivalent to the sum of the inactivation observed after AE and HPP as a single treatment for all *L. monocytogenes* strains, except for 12MOB089LM, which was 1.0 log lower than the theorical inactivation of AE plus HPP, suggesting a possible cross-protection effect of acid exposure against HPP effects. When the enumeration was carried out 24 h after treatments, 12MOB047LM and Scott A strains showed 1.3 and 0.8 log lower inactivation, respectively, compared with the sum of the inactivation obtained for AE and HPP as single treatments, while for CTC1034 and 12MOB089LM the effect of AEHPP was in agreement with the additive effect of each treatment.

**Figure 3:**
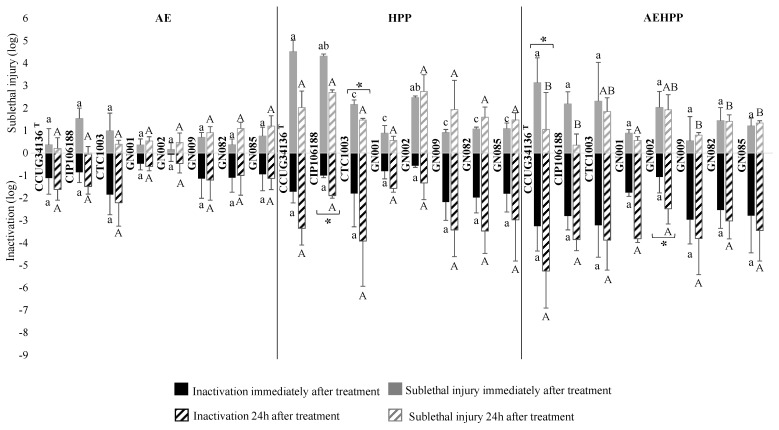
Mean logarithmic reduction (black) and sublethal injury (grey) for each strain of *Salmonella* after acid exposure (AE), high-pressure processing (HPP) and both (AEHPP) treatments of apple puree sampled immediately (solid) and 24 h (striped) after treatment. The standard deviation is shown with error bars. The different letters show statistically significant (*p* < 0.05) differences between strains within each treatment (lower case for inactivation immediately after treatment, and upper case for inactivation 24 h after the treatment). Asterisks show statistically significant (*p* < 0.05) differences between sampling immediately and 24 h after treatment.

Regarding *Salmonella* (Figure 3), the average inactivation observed among the strains originally included in the cocktail was similar, and more strains were subsequently included in the study. However, the inactivation recorded within the performed trials was quite variable among the independent trials for all three treatments (AE, HPP and AEHPP) applied, as shown by the standard deviation bars. Some strains, e.g., GN001, GN002 tended to be more resistant to all treatments compared with others (e.g., CTC1003, CCUG34136^T^) with 2.2 log units difference on the average inactivation. However, such differences were not statistically significant (*p* > 0.05). The inactivation increased when the enumeration was carried out 24 h after the treatments; however, the statistical significance of the difference with the inactivation immediately after the treatments could only be confirmed for a few strains (i.e., CIP106188 for HPP and GN002 for AEHPP).

Regarding the combined effect of acid exposure before HPP (AEHPP treatment), the inactivation measured immediately after treatment agreed with the theoretical sum of the inactivation obtained for the single treatment (additive effect) for all strains. When enumeration was performed 24 h after the AEHPP treatment, the inactivation was lower than the sum of the mean inactivation with the corresponding single treatments for some *Salmonella* strains (CTC1003, GN082 and GN085), while higher for others (e.g., GN001 and GN009).

**Figure 4:**
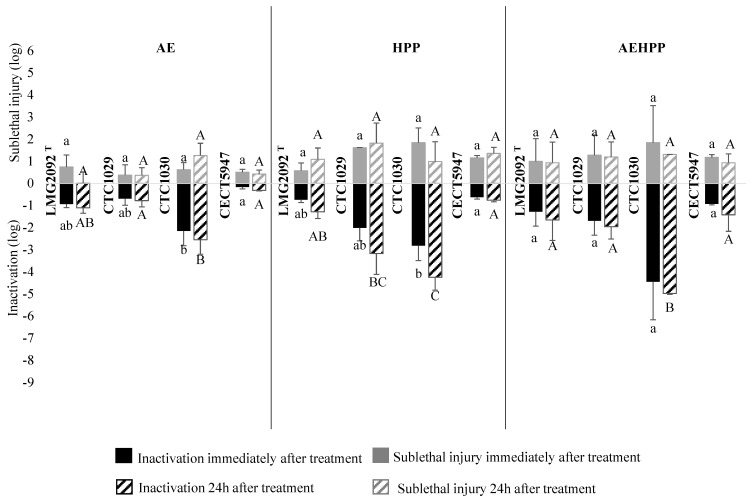
Mean logarithmic reductions (black) and sublethal injury (grey) for each strain of *E. coli* after acid exposure (AE), high-pressure processing (HPP) and both (AEHPP) treatments of apple puree sampled immediately (solid) and 24 h (striped) after treatment. The standard deviation is shown with error bars. The different letters show statistically significant (*p* < 0.05) differences between strains within each treatment (lower case for inactivation immediately after treatment, and upper case for inactivation 24 h after the treatment).

In general, *E. coli* (Figure 4) was the most resistant species among the evaluated bacteria, especially the stx-negative O157:H7 strain CECT5947. Irrespective of the sampling time (which did not result in a statistically significant influence), the inactivation due to AE ranged from 0.2 log in CECT5947 to 2.2 log in CTC1030 (*p* < 0.05). The strains also showed significant (*p* < 0.05) differences in the HPP inactivation between the most piezo-resistant strain (CECT5947, with 0.6 ± 0.1 log reduction) and the most piezo-sensitive (CTC1030, with 2.8 ± 0.7 log). The combined AEHPP treatment resulted in an additive inactivation effect for the most resistant strain, CECT5947 (ca. 1 log reduction in total), while for the most sensitive strain, CTC1030 (ca. 4 log reduction), the inactivation was lower than the theoretical sum of single treatments, suggesting a cross-protection.

### 3.3. Sublethal Injury Associated with HPP and Acid Exposure

Sublethal injury associated with AE, HPP and AEHPP treatment of *L. monocytogenes*, *Salmonella* and *E. coli* in apple puree is shown as grey bars on Figure 2, Figure 3 and Figure 4, respectively.

Strains of *L. monocytogenes* did not show statistical differences (*p* > 0.05) on the sublethal injury rate immediately after AE treatments (i.e., on average, 0.6 ± 0.6 log). After 24 h-delayed enumeration the sublethal injury tended to increase, except for 12MOB047LM, which decreased significantly (*p* < 0.05). In HPP puree sampled immediately after treatment, the evaluated strains showed few significant differences (*p* < 0.05) regarding sublethal injury, which ranged from 0.6 ± 0.2 log (12MOB047LM) to 1.7 ± 0.2 log (12MOB089LM), tending to decrease after 24 h of the treatment. In contrast, similar sublethal injury (*p* > 0.05) was found for all the strains after AEHPP treatment for both immediate (0.5 ± 0.2 log) and 24 h-delayed (1.2 ± 0.9 log) enumeration.

*Salmonella* sublethal injury after AE was rather limited (0.7 ± 0.5 log, without differences between strains), while it increased to 0.9–4.5 log after HPP with some differences depending on the strain; i.e., CCUG34136^T^ (4.5 ± 0.5 log) and CIP106188 (4.3 ± 0.1 log) were the most sublethally damaged strains, while GN009, GN082 and GN085 showed a sublethal ratio close to or below 1 log. AEHPP resulted in a variable sublethal damage ratio between independent trials, which hampered the detection of statistically significant differences between strains (*p* > 0.05, the average range being 0.5–2.1 log). Delayed sampling only affected (reduced by more than 0.5 log, *p* < 0.05) the sublethal injury of CTC1003 after HPP and CCUG34136^T^ after AEHPP, due to pressure-damaged cells that may have died.

The sublethal injury observed for *E. coli* was not dependent on the strain or sampling time (immediate or delayed) (*p* > 0.05) (Figure 4), which was 0.5 ± 0.5 log, 1.3 ± 0.6 log and 1.2 ± 0.7 log after AE, HPP and AEHPP, respectively.

The correlation between the magnitude of the inactivation and the ratio of sublethal damage was assessed considering the results of all treatments for each specific strain (Table 1). No common trend within species or within strains could be observed. For some specific strains, such as *L. monocytogenes* CTC1034, *E. coli* CTC1029, LGM2092 ^T^, CECT 5947 and *Salmonella* GN001, GN082, and GN085 a clear and significant linear relationship was established, i.e., the higher the inactivation the higher the sublethal injury. In contrast, for *L. monocytogenes* ScottA, higher sublethal injury was observed at lower inactivation magnitude.

## 4. Discussion

The resistance of microorganisms to HPP is associated with the cell structural complexity, eukaryotic microorganisms (molds and yeast) being more piezo-sensitive than prokaryotic bacteria. Within vegetative bacteria, Gram-positive are often more resistant than Gram-negative [26], though it might also depend on the food matrix and particularly on the strain [3]. Understanding the variability of HPP inactivation within different species and strains is a critical step before validating non-thermal technologies for food preservation. The most resistant microorganisms of public health significance among those that are likely to occur in the food of interest have to be considered [16]. In low-acid food of animal origin, *Salmonella* and *L. monocytogenes* are usually the target pathogens and have been reported to be more resistant than *E. coli* [7]. In the current study dealing with acid fruit puree, *E. coli* strains showed higher piezo-resistance compared to those of *Salmonella* and *L. monocytogenes*. In this line, Podolak et al. [3] reported that Gram-positive bacteria (*L. monocytogenes*) were less resistant to HPP than Gram-negative bacteria (*E. coli* and *Salmonella* spp.) in pressurized apple juice treated at 550 MPa for 1 min at 5 and 20 °C. In other studies dealing with Gram-negative bacteria, *Salmonella* was more piezo-sensitive than *E. coli* in grape juice (pH 3.39) [12,27]. Simpson [28] also reported that *E. coli* O157:H7 was more resistant compared to *L. monocytogenes* and *Salmonella* in acidic whey beverages (pH 3.8) treated with HPP at 300–500 MPa for 2 to 8 min at 25 °C. Specifically, in apple puree (pH 3.8), *L. monocytogenes* and *Salmonella* Typhimurium were completely inactivated (greater than 6-log reduction) at 300 MPa for 5 min, while *E. coli* O157:H7 was still detected (ca. 5-log reduction) after a more intense treatment at 300 MPa for 7 min [29].

Fruit acidity and HPP are stress factors that can cause lethal (inactivation) effect on the cells, but also sublethal injury, which may compromise their ability to recover and grow in certain culture media during enumeration or in foods during their shelf-life [30]. Therefore, the impact of the type of enumeration agar on sublethally damaged cells, e.g., after exposure to harsh conditions of the food matrix and/or the application of a preservation treatment, should be considered, to avoid over- or under-estimation of the microbial inactivation. Specifically, the ability of a cell to grow in a culture medium depends on one hand on the extent of sublethal injury suffered by the cell, which depends on the intensity of the treatment and specific characteristics of the cell (i.e., species, strain and physiological state) and on the other on the inhibitory capacity of the medium, as injured cells may not be able to form colonies under non-optimal conditions [5]. Accordingly, in the present study, the magnitude of inactivation of *L. monocytogenes*, *E. coli* and *Salmonella* strains was dependent on the type of enumeration agar. Generally, a slightly higher inactivation appeared after some treatments (e.g., *L. monocytogenes* after AE or *E. coli* after HPP and AEHPP) when enumerating with a selective agar (TS, S, and SO) compared to when using the nutrient-rich TSYAE, which allowed the recovery of injured cells. In agreement with this, other studies evaluating the impact of plate count agars on the enumeration of pathogens have also shown lower counts in selective agars after high-intensity heat-based treatments [31,32]. In this regard, Teo et al. [33] studied HPP inactivation of an *E. coli* cocktail at 615 MPa for 1 min at 15 °C in orange juices, and reported higher inactivation when measured in a selective (5-log reduction in MacConkey sorbitol agar) than in a non-selective medium (2.2-log reduction in TSAYE). Considering that injured-cell recovery depends on the matrix characteristics (enumeration media and/or food), the knowledge of the impact of the culture media on the viability and colony-forming capacity of target pathogens is relevant when performing validation studies, to quantify the lethality of processing and preservation treatments. Therefore, to avoid an overestimation of the inactivation effect of AE and the efficacy of HPP, the selected agars for the subsequent study to characterize the inactivation and sublethal injury of individual strains of *L. monocytogenes*, *Salmonella* and *E. coli* were TSAYE (T) and TSAYE + 4% NaCl (TS).

Moreover, to better understand the impact of food acidity on cell recovery, enumeration was performed immediately and 24 h after the application of each treatment. The storage of samples up to 24 h under refrigeration (4 °C) between sampling and the actual microbiological analysis is an accepted practice for food product-testing purposes, to maintain sample integrity and minimize bacterial growth before analysis during the transport of the samples from the sampling site to the laboratory [34]. During this short time, storage cells may recover from the stress caused by a processing technology such as HPP and be able to form colonies in the agar plate. In low-acid food matrixes (e.g., raw pet food pH = 6.8), Serra-Castelló et al. [7] found 1-to-2-log unit higher *Salmonella* counts when the enumeration was performed 24 h after HPP (at 600 MPa for different times, 1–10 min), resulting in a lower inactivation compared with the measurement carried out immediately after HPP. However, when the pet food was acidulated with lactic acid (to pH 5.7), the cell recovery was hampered, and the same level of inactivation was recorded for the two sampling times (immediately after and 24 h after the HPP). In our study, the more acidic pH not only prevented the recovery of the cell, but contributed to reducing the viability of the cells surviving the HPP.

The pH is among the most significant factors acting as an antimicrobial hurdle inhibiting the growth of bacteria in foods and also enhancing the lethal effects of physical treatments such as HPP. Accordingly, in the present study, higher HPP inactivation of the three pathogens was achieved in apple (more acid) than in banana–apple. Previous studies demonstrated that vegetative foodborne pathogens are more susceptible to HPP at a lower pH. Gouvea et al. [35] observed a significant impact of pH on the inactivation of *Salmonella* spp. in açaí juice treated at 400 MPa for 3 min. Specifically, at pH 4.5, Salmonella spp. decreased by 3.5 log, while at pH 4 it reached more than an 8-log reduction, to levels below the limit of detection (0.7 log CFU/mL). Xu et al. [36] found that *S. enterica* and *L. monocytogenes* were more sensitive to HPP in orange juice (pH 3.46) than tomato juice (pH 4.11). The effect of pH is attributed to the damage to the maintenance of pH homeostasis, cell membrane integrity and fluidity, metabolic regulation, and macromolecule repair [5,29,37].

As described by the hurdle technology concept in food preservation, the application of an intelligent combination of several stress factors or antimicrobial hurdles improves food safety because the foodborne pathogenic microorganisms are unable to overcome them [38]. The hurdles can have additive effects, as shown in most of the cases in our study, when combining acidic exposure and HPP. Other strains showed a magnitude of log reduction lower than the theoretical sum of the single effects, which could suggest a cross-protection effect exerted by the acid exposure prior to HPP. It has been reported that exposure of microorganisms to harsh conditions may trigger stress responses that confer protection against the subsequent stresses, which represent a potential increased risk to food safety [39]. As an example, Wemekamp-Kamphuis et al. [40] found that *L. monocytogenes* EGD-e cells exposed to acid (pH 4.5) for 1 h in brain heart infusion (BHI) media developed cross-protection effect against HPP (250–350 MPa for 8 min) and about 2-log lower inactivation was recorded compared to non-exposed cells. Therefore, from the practical point of view, given the fact that acidic matrixes enhance HPP inactivation, the application of HPP would be better applied immediately after packaging. In this way, the possibility that bacteria develop acid-induced cross-protection mechanisms against HPP, thus reducing the efficacy of HPP, will be minimized.

Microorganisms may be present as sublethally injured cells when they survive an inactivation treatment [25]. In the present study, *L. monocytogenes* presented less sublethal injury compared to the other Gram-negative bacteria (*E. coli* and *Salmonella*). Sublethally injured cells have been previously described in pressurized acidic products such as fruit and vegetables juices. Nasilowska et al. [41] confirmed that the application of 300 MPa for 5 and 10 min caused 0.72 log and 0.58 log of sublethal injury of *L. innocua* (CIP 80.11^T^) in beetroot juice (pH 3.98). In pressurized tomato juice (250 MPa for 5 min) *L. monocytogenes* showed 2 and 1.5 log of sublethal injury, immediately and 24 h after HPP, respectively [10]. Furthermore, Jordan et al. [10], also studied the ratio of sublethally injured cells of *E. coli* O157 in tomato juice (pH 4.1) after a pressurization treatment of 300 MPa for 5 min and reported 0.29 and 1.73 log of sublethal injury, immediately and after 24 h of storage at 4 °C after HPP, respectively. Similar results were observed in the current study, as strains of *E. coli* showed an average of 1.2 ± 0.4 log and 1.2 ± 0.3 log of sublethal injury, respectively, in HPP and AEHPP treatment. Sublethally injured cells depend on several factors such as the treatment parameters (i.e., pressure, time, temperature) and the specific characteristics of the microorganism (resistance to acidity, temperature, pressure) and no clear relationship with the magnitude of inactivation can be generalized. Therefore, sublethal injury should be considered (i.e., quantified) to avoid an overestimation of the treatment, and they entail consequences at the food safety level, especially in foods for infants.

An additional step to account for sublethally damaged cells is sampling 24 h after HPP treatment. Delayed sampling 24 h after HPP involves sublethally injured cells being submitted to unfavorable conditions (acidity of the matrix), which can impair their recovery. In the studied apple puree, when sampling 24 h after treatment no recovery was observed, as inactivation increased by more than 0.5 log in all studied treatments. The viability loss of HPP-damaged cells has been reported in other studies [35,42,43]. For instance, Pagán et al. [43] found that *E. coli* O157 was more acid-sensitive after pressurization, due to the loss of protective or repair functions or the loss of pH homeostasis. Also, Gouvea et al. [35] found a decrease in *L. monocytogenes* and *Salmonella* spp. in pressurized açaí juice (300 MPa/3 min) during the subsequent refrigerated storage, not allowing the sublethally injured cells to recover, which can explain the additional reduction observed with delayed sampling.

## 5. Conclusions

The effectiveness of HPP as a microbial lethality treatment in fruit puree was influenced by the bacterial species, and particularly the specific strain, the intrinsic factors (i.e., acidity) of the product and the exposure period before HPP, but also by factors related to verification procedures, such as the sampling time (immediately or 24 h after HPP) and the enumeration agar. Accordingly, all these factors should be taken into account when designing HPP treatments, performing product-specific validation studies and setting the verification procedures. In addition, for performing validation studies of HPP in fruit purees, *E. coli* should be the target pathogen, as it is the most piezo-resistant bacteria, highlighting the relevance of the O157:H7 serovar (i.e., CECT5947 strain). Enumeration agar has an impact on the viability of the survivors and should be carefully considered when comparing different studies, as well as when evaluating the lethality of acidity and/or HPP as control measures within the food-safety management system, as some selective media hamper the formation of colonies by sublethally injured cells, which would result in an overestimation of the lethality of a treatment. Delayed sampling in the verification procedures would be more indicative of the eventual HPP effect in acid matrixes. Further studies are required to better understand the impact of acid exposure before HPP at different pressure levels.

## Figures and Tables

**Table 1:** Correlation *^a^* between the inactivation (log *N*/*N_0_*) and the sublethal injury ratio for each bacterial strain assessed.

Bacteria	Strain	Slope	R^2^	*p*-Value
*L. monocytogenes*	12MOB047LM	−0.51	0.327	0.052
	12MOB089LM	−0.02	0.001	0.910
	CTC1034	−0.29	0.387	0.031
	Scott A	0.30	0.874	<0.001
*E. coli*	CTC1029	−0.70	0.829	<0.001
	CTC1030	0.11	0.032	0.579
	LGM 2092^T^	−0.92	0.507	0.009
	CECT 5947	−0.49	0.351	0.042
*Salmonella*	CCUG34136^T^	−0.01	0.000	0.966
	CIP106188	0.71	0.254	0.095
	CTC1003	−0.27	0.172	0.180
	GN001	−1.09	0.697	0.001
	GN002	−0.36	0.167	0.188
	GN009	−0.22	0.193	0.154
	GN082	−0.20	0.625	0.002
	GN085	−0.34	0.596	0.003

“*^a^*”: linear correlation was assessed considering all treatments for each bacterial strain, i.e., after high-pressure processing (HPP), acid exposure (AE) or the combination of acid exposure and high-pressure processing (AEHPP), measured just after the treatment or after 24 h.

## Data Availability

The original contributions presented in the study are included in the article, further inquiries can be directed to the corresponding author.
